# Secure medical image transmission using deep neural network in e‐health applications

**DOI:** 10.1049/htl2.12049

**Published:** 2023-07-19

**Authors:** Ala Abdulsalam Alarood, Muhammad Faheem, Mahmoud Ahmad Al‐Khasawneh, Abdullah I. A. Alzahrani, Abdulrahman A. Alshdadi

**Affiliations:** ^1^ College of Computer Science and Engineering University of Jeddah Jeddah Saudi Arabia; ^2^ School of Technology and Innovations University of Vaasa Vaasa Finland; ^3^ School of Information Technology Skyline University College University City Sharjah Sharjah United Arab Emirates; ^4^ Department of Computer Science, Collage of Science and Humanities in Al Quwaiiyah Shaqra University Shaqra Saudi Arabia; ^5^ Department of Information Systems and Technology, College of Computer Science and Engineering University of Jeddah Jeddah Saudi Arabia

**Keywords:** chaotic, confusion, deep neural network, diffusion, encryption, randomness, region of interest

## Abstract

Recently, medical technologies have developed, and the diagnosis of diseases through medical images has become very important. Medical images often pass through the branches of the network from one end to the other. Hence, high‐level security is required. Problems arise due to unauthorized use of data in the image. One of the methods used to secure data in the image is encryption, which is one of the most effective techniques in this field. Confusion and diffusion are the two main steps addressed here. The contribution here is the adaptation of the deep neural network by the weight that has the highest impact on the output, whether it is an intermediate output or a semi‐final output in additional to a chaotic system that is not detectable using deep neural network algorithm. The colour and grayscale images were used in the proposed method by dividing the images according to the Region of Interest by the deep neural network algorithm. The algorithm was then used to generate random numbers to randomly create a chaotic system based on the replacement of columns and rows, and randomly distribute the pixels on the designated area. The proposed algorithm evaluated in several ways, and compared with the existing methods to prove the worth of the proposed method.

## Graphical abstract

This study proposes a deep learning‐based secure medical image transmission in e‐health applications

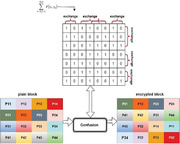

## 1 INTRODUCTION

In our current era of information technology, the rapid growth of the Internet, especially in electronic health care, has made electronic health care feasible and widespread. Electronic health care is considered one of the systems based on the Internet in a way that makes the patient able to contact the specialist doctor who is able to diagnose. Medical images are stored and sometimes processed, and then sent to the Internet later. During storage, some secret data can be added. The addition of secret data is often necessary but is sensitive for both parties. Therefore, the best way to preserve the privacy of patients, whose information may be highly confidential and sensitive, is to encrypt the data in a way that it is available only to authorized persons [1]. There are some characteristics that distinguish medical images, including redundancy, high correlation between image pixels, and huge size of data. Unlike the ordinary images, encryption in medical images requires a special effort related to the speed and accuracy in extracting data. However, it is sometimes not suitable for securing large medical images. It is therefore necessary to secure the algorithms that process medical images against attacks [2]. A random number generator is usually used to generate a string of numbers in a schematic sequence rather than a logical one used for encryption. The more random the number that is generated, the more it helps in encryption, and is more feasible. In this regard, chaos systems are used to generate and design pseudo‐random numbers in order to generate a strong encryption key [3]. Some of the techniques are distinguished from other technologies in several aspects, including in terms of the power of generating initial random numbers, the reactions of the numbers, the amount of their sensitivity to the initial conditions, the long frequency, and the availability of space for the large encryption keys. Accordingly, this paper attempted to combine two methods of chaotic system and key optimization which will provide great performance in terms of security and speed. In addition to the rigor and strength of the algorithm, the contribution to an efficient algorithm must be the encryption process. Most of the algorithms are not efficient in real time because of the length of the algorithm sequence and the performance is not efficient to be online [4]. Algorithms are often vulnerable to attacks from external programs, especially in the case of online transmission. With regard to the development of the current devices and their wonderful performance, it contributed to the improvement of algorithms, especially in terms of random generation of numbers. In general, the performance of the devices is in two main aspects: increasing the frequency of the device (processor) speed and optimizing the use of a specific processor. In terms of hardware implementation, there are two options, ASIC and FPGA; the first is somewhat costly, and the second is a promising solution. FPGI is efficient in that it allows the designer to use basic programmable logic elements in designing and creating intelligent algorithms, but they are generally expensive in terms of design in general. As for the other type, it can be developed to be the same type of performance at a lower cost, but with tremendous efforts. The first type is capable of executing at the required speed and means processing hardware more than software.

Deep Learning (DL) and Artificial Intelligence (AI) have advanced rapidly in recent years. Artificial intelligence techniques have played very important roles in the field of medical images [5], such as processing and diagnosis by computer, and performance of all operations on images such as interpretation, merging, recording, and segmentation. Most of the images come from directed devices with radiation, and therefore, they are retrieved and analyzed using deep learning to extract information from the image, and the information is represented effectively. Deep learning algorithms help doctors make accurate decisions in diagnosing diseases quickly and efficiently, and this could prevent diseases in a timely manner. Artificial intelligence techniques help doctors understand how to analyze images, the differences between them, and the real causes of the disease. Artificial intelligence techniques include a lot of methods such as support vector machine (SVM) [6, 7], neural networks (NN) [8], K‐nearest neighbor (KNN) [9], and other algorithms related to deep learning like convolutional neural networks (CNN), recurrent neural networks (RNN), and long short term memory (LSTM) [10], and the extreme learning model (ELM) and generative adversarial networks (GANs), which are limited in processing natural images and require a long time for analysis and more time for processing features. These algorithms are fed with raw data in analyzing the data as a whole, followed by the required classification. Learning algorithms try to learn many levels of abstraction and representation, and glean information from a large set of images that often exist in a standard database. These images show the desired behaviour of the data. Despite the discovery that was made in diagnosing diseases through medical imaging according to traditional methods, which showed accuracy in diagnosis over decades of time. Therefore, the development of deep learning has made a breakthrough in the performance of algorithms and the accuracy of work. At present time, speed and accuracy have been among the most sought‐after important factors. Meanwhile, deep learning algorithms have proven their efficiency in many areas such as speech recognition, lip reading, text recognition, accurate computer diagnosis, face recognition and effective drug discovery.

The motive of this study was to develop a method for processing medical images using deep learning technology, controlling the number of hidden layers in the neural network using the highest effect factor, and processing and classifying the extracted features in a new way to diagnose diseases and heal the image in a way that protects the information from threats such as hacking.

The contribution of this manuscript is to find a chaotic system that is not detectable using DNN algorithm and adaptation of the deep neural network by the weight that has the highest impact on the output, whether it is an intermediate output or a semi‐final output.

The remainder of the manuscript has been arranged as follows: Section 2 is related to previous studies related to the subject and the most important studies in this field. The subject methodology is presented in Section 3. Then, the results are presented in Section 4. Finally, the conclusions are presented in Section 5 of this research.

## 2 RELATED WORK

Many previous studies have dealt with Security images in general [11], and medical images in particular [12]. The security of data and images is related to the strength of the proposed algorithm. Many algorithms have been proposed in the literature and often rely on robust random key generation [13]. A method of generating a secure key with reduced latency and based on the cardiogram in encryption [14] has been proposed. An improved method was proposed by [15] to heal the medical image using Fibonacci, which had the effect of scattering and hiding the information of the medical image [16]. The AES algorithm method was used to generate random numbers [17] from electrical impulse generators to increase the security of the resulting image. A dynamic cipher system based on state estimation has been proposed by [18] which is the basis for cipher key generation. The level of challenge was raised in relation to generating the highest randomness in relation to the initial key, which constantly changes with work and time [19], and it had an effective impact on the security of the medical image transmitted between the parties.

Exploring the chaotic system had an impact on the safety of the medical image [20] and the exploration of the usefulness of linear systems, which deals with the basic elements of coding such as sensitivity, predictability, pseudo‐randomness and certainty [21]. Seed generation by the artificial intelligence system is a method proposed in order to increase the randomness of the data distribution in medical images, which is difficult to trace the data [22], and it is a method based on complete randomness and has proven its effectiveness through graphing and linear results. A method adopted by current researchers [23] depends on a new method that has proven its worth through evaluation, which relies on a hybrid algorithm to generate chaos in the pixel distribution of the image again [24], and the chaotic sequences are difficult to track inside the image, only through the encryption key through which it is retrieve data [25].

**FIGURE 1:**
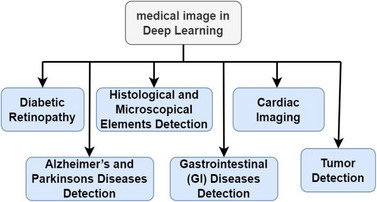
type of medical image detection by deep learning technique.

Other researchers worked to cancel the relationship of pixels in the image that were preserved by certain equation, and this equation works to track the effect of pixels in the image and build a path that can be traced by the encryption key. Artificial intelligence techniques were used in the encryption of medical images in order to increase the security of data in them [26], and many researchers obtained unexpected results when using algorithms based on artificial intelligence such as ANN, CNN, and SVM, decision tree, NN, and others such as KNN [27]. Many medical image diagnostic tasks require research and new ways to identify abnormalities and changes to images over time. Deep learning algorithms came to open up many horizons in this regard. Many image resources are processed in deep learning techniques, which are sourced from three main types (X‐rays—CT—and MRI scan). Most of the types of diseases diagnosed using deep learning are shown in Figure 1.

i. Diabetic retinopathy: The manual process of diagnosing and detecting diabetic retinopathy (DR) is difficult and takes a long time, as this disease does not show early symptoms, and so, the doctor needs a picture of the bottom of the retina. Accordingly, deep learning has proven its accuracy in working on this type of image. A deep learning algorithm was used in the Deep Convolutional Neural Network (DCNN) from the eye image archive to classify the signals of the moderation [27]. The deep learning algorithm was trained on standard dataset (EyePACS‐1) collected from 847 patients, and the accuracy and sensitivities exceeded 95% [28]. And another study used DNN on different dataset (Kaggle fundus) to diagnose bleeding, secretions and aneurysms [29].
ii. Histological and microscopical elements detection: In histological analysis, the cell and surrounding tissues are studied. The changes that occur in it reflect the characteristics and features through which the disease can be diagnosed. Imaging and colouring of cells in dermatology give a clear picture of the disease. Therefore, to increase the accuracy of diagnosis, artificial intelligence algorithms such as DNN are used. Recently, many researches related to this type of disease have been published, and DNN applications have been used to diagnose cancer cells in the colon [30, 31]. Another study was conducted on thoracic lymph nodes and interstitial lung disease using CNN algorithm. There are many studies that used CNN in the early diagnosis of breast cancer by classifying features extracted from medical pictures [32]. A standard dataset was used to train the RNN algorithm by 75%, and the results showed its effectiveness in detecting the disease early and in limiting its spread.
iii. Gastrointestinal (GI) diseases detection: Mock processing through deep learning plays a vital role in analyzing diseases and in helping doctors in providing accurate and efficient treatment, owing to the advancement of computer science, computer analysis and the science of image processing, which are obtained from X‐rays or magnetic resonance imaging (MRI). Accordingly, the DCNN method was used to detect haemorrhage in images from capsule endoscopy [33]. Additionally, a study was carried out using fully supported and fully stacked FCN networks compatible with LSTM by dividing big data into small data for ease of obtaining characteristics [34]. In another study, a set of features was extracted by the hybrid method and classified using CNN to detect digestive diseases in the image of MRI [35]. In a related study, a rapid feature extraction method using CNN technique was presented to detect inflammatory gastrointestinal diseases in WCT videos, and the extracted features were classified using SVM [36].
iv. **C**ardiac imaging: Deep learning provided an excellent method for imaging the heart, and in particular for measuring calcium scores. MRI images are common in this field [37]. SVM was used to classify the features extracted from the image and find the appropriate diagnosis [38, 39]. The diagnosis of the heart was done using features that enter into more than one of the hidden layers, which in turn gives an output and a line that is taken into account in choosing the accuracy of the result in the NN algorithm [40].
v. Tumour detection: Abnormal cell growth in one place is called a tumour. There are two types of tumour; one is non‐cancerous (benign tumour) and the other is cancerous (malignant tumour). A study by [41] employed a method for diagnosing a tumour from a mammogram in a database containing 482 images, after removing noise using a median filter. Many researchers in the literature have used the famous SVM classifier to classify the features extracted from mammograms to diagnose benign or malignant tumours. The CNN algorithm was fed with specifications extracted from radiographic images, measuring the amount of clustering in each part of the image, and relying on it in the classification to diagnose the disease [42].
vi. Alzheimer's and Parkinson's diseases detection: This disease is considered a neurological disorder associated with a pro‐faulty decrease in motor accuracy. This disease is associated with the breakdown or death of dopaminergic neurons. Alzheimer's disease can be diagnosed by clinical images and by measuring the shift in the fixed features in the CT image. The deep Boltzmann machine (DPM) was used to measure the additional features and find out the distortions of the 3D magnetic resonance images [43]. 3D image was explored by [44] to investigate Alzheimer's disease by extracting good features of the image and classifying them using CNN. A dataset was used CAD Dementia for MRI and satisfactory results were obtained for people over the age of 75 years. Also, another group of authors used RMI brain images to detect whether the brain is healthy or of Alzheimer's, and the result was 98% in accuracy for training the data on images of basal standard data [45].

## 3 PROPOSED METHOD

The work methodology in general consists of two main parts, whereby the first is to maintain data security in the medical image by improving its encryption method, and the second is to classify the features extracted from the image using deep learning and DNN algorithm. In encryption in general, there are two stages, the contribution to the proposed method lies in the two main parts of the work: confusion and diffusion. For confusion we choose pixel position and sub‐blocks randomly by helping of DNN algorithm in additional to changing the value of pixels controlled also by the same algorithm. Encryption is the process of hiding image information so that it is secure and no one can see it without the encryption key. The encryption is based on the irregular scattering of data in relation to the outside world, and it cannot be rearranged except with the presence of the encryption algorithm. The proposed method is based on a chaotic system that extends in two stages: The region of interest (ROI) and the pixels in that region. First, the random key is generated, and chaos systems are very useful in this case. The entropy of the image as well as its statistical behaviour in randomness are handled. The Henon Map method was used to generate the key because of the ideal behaviour of this algorithm as well as it is scalable and compatible with many methods. The following Equation (1) can define the key generation:

(1) $$\mathrm{initial}\;\mathrm{key}\;=\left\{\begin{array}{c} x_{n+1},\;\;\;\;1-ax_{n}^{2}+y_{n}\\ \;\;\;\;\;\;\;\;\;\;\;\;\;\;\;\;\;\;y_{n+1},\;\;\;\;bx_{n}\; \end{array}\right.$$

where *x* and *y* are two variables and *a* and *b* are two parameters that satisfy the chaotic behaviour such as *a = *1.4 and *b = *0.3, while *n* is iteration numbers, in Henon initial state that starts with *x*_0_ and *y*_0_ to initialize the key.

**FIGURE 2:**
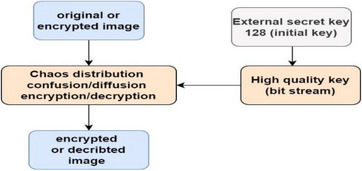
General illustration of proposed encryption system.

The system in general starts with generation of key of 128, followed by the formation of bit stream of random sequence, and then the initiation of encryption with variable iterations as shown in Figure 2.

The deep learning algorithm uses the proposed method in the process of encryption the medical image from the process of reading the images from the standard dataset and then applying all the procedures to it from segmenting it into blocks and then changing the pixel values. As explained in Algorithm 1

ALGORITHM 1 **General proposed stage for image encryption using DNN.**

**Table:** 

1‐ **Read** images from dataset 2‐ **For** all image **do** 2.1 **Preprocessing** given image 2.2 **Extract** features from image 2.3 **Creat**e Neural Network 2.4 **Determine** effective parameters in the network 3‐ **Update** hidden layers and nodes according certain parameters 4‐ Confusion process 4.1 **Use** DNN to select partition of image 4.2 **For** each partition use DNN to scrambling 4.3 **Update** cypher key 5‐ Diffusion process 5.1 **While** not EOI Do 5.1 **Move** pixels into vector 5.2 **Use** DNN to change pixel value (vertically and horizontally) 5.3 **Update** cypher key 6‐ Save the encrypted image to a file or transmit it through a secure channel for storage or further processing 7‐ Return to step 2

**FIGURE 3:**
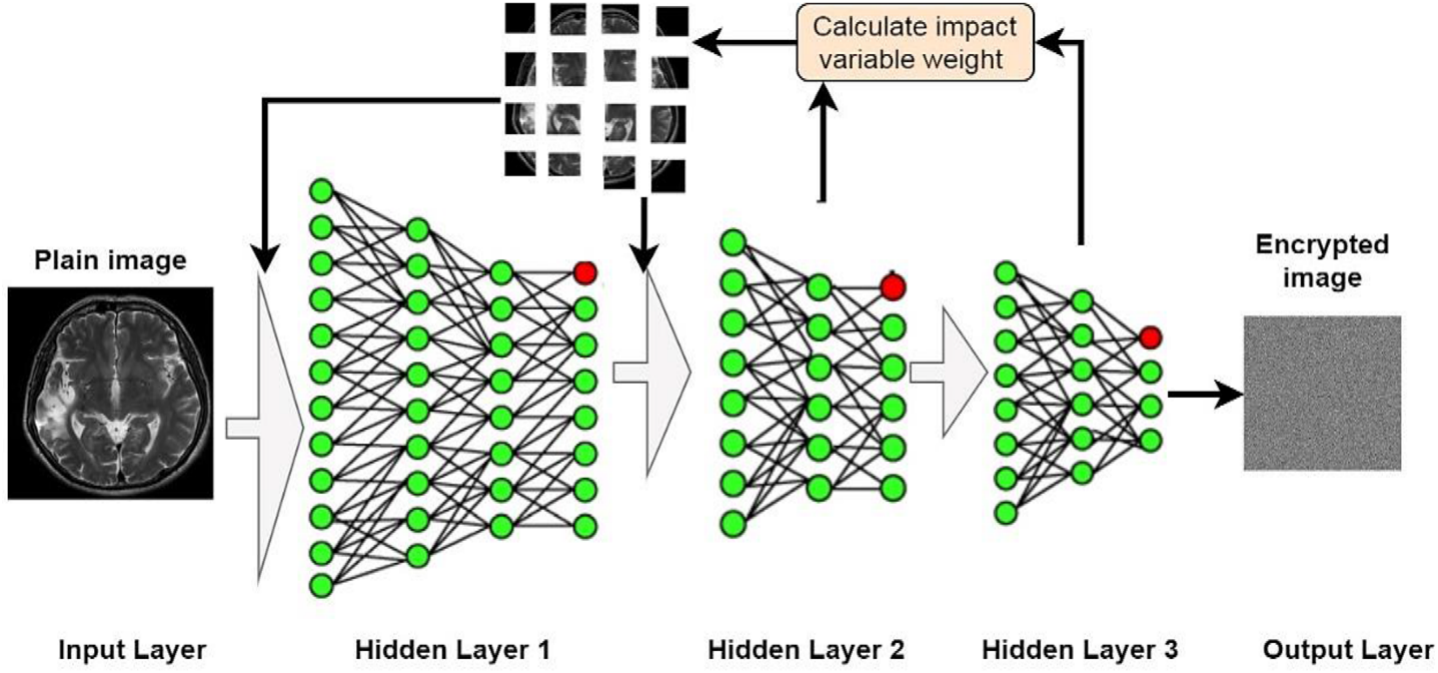
Behaviour of proposed image encryption within deep learning algorithm.

In order to generate a high‐quality key, which is the basis for the encryption process, some variables can be suggested; these variables are in the form of parts that can be interlocked with deep learning. The variables can be described as follows:

(2) $$X\;h_{0}=\frac{\left(k_{1}⊕k_{2},⊕\text{…},⊕k_{8}\right)}{2^{8}}$$

(3) $$Y\;h_{0}=\frac{\left(k_{9}⊕k_{10},⊕\text{…},⊕k_{16}\right)}{2^{8}}$$

where $K_{i}=k_{1}\;|k_{2}|k_{3},\text{…},{|k}_{16}$ are the sequence of iteration for each layer in neural system. The encryption key uses itself in the initial cycles as soon as the hidden layers are changed in deep learning. The value of key 16 is better than its analogues to create a chaotic context added to the design of nodes in the proposed layers in the neural network system. Then, the statistic behaviour of the random system can be described in Figure 3.

The random number remains in constant change with each cycle in random sequence. This allows the conversion of the number to the appropriate chaotic state of the system. A key consisting of 128 works on a highly random statistical behaviour can be described by the following equations:

(4) $$XH\;=\left(Xh_{i}\times 10^{12}\right)\;\mathrm{mod}\;2^{16}$$

(5) $$YH\;=\left(Yh_{i}\times 10^{12}\right)\;\mathrm{mod}\;2^{16}$$

**FIGURE 4:**
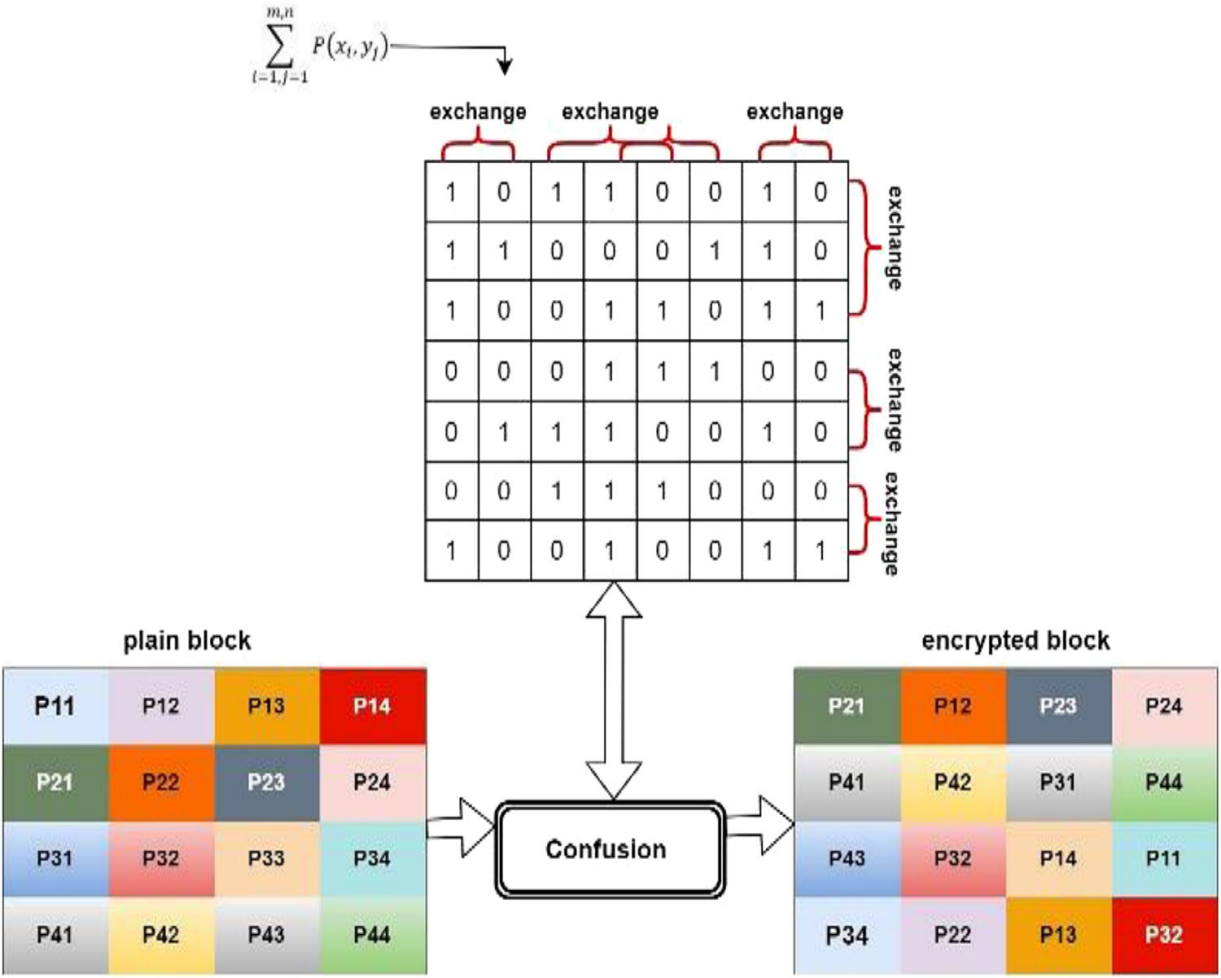
Confusion process in proposed method.

In encryption in general, there are two stages. The first stage is the confusion stage, in which the locations of the pixels in the image are changed. In this regard, the locations are of two types. First, changing the columns according to certain equation and second is changing the rows to increase the randomness of secret image. The process of changing, with respect to rows and columns, increases the security of the medical image, in addition to changing the dependence of parts of the images cut from the original image through deep learning. Figure 4 can be referred.

The second stage of image encryption is called diffusion, and through it, the pixel values of the image are changed, and thus noise is generated. To achieve encrypted image, the OR operation is performed between the pixel value, the K key, and the scrambled image vector.

**FIGURE 5:**
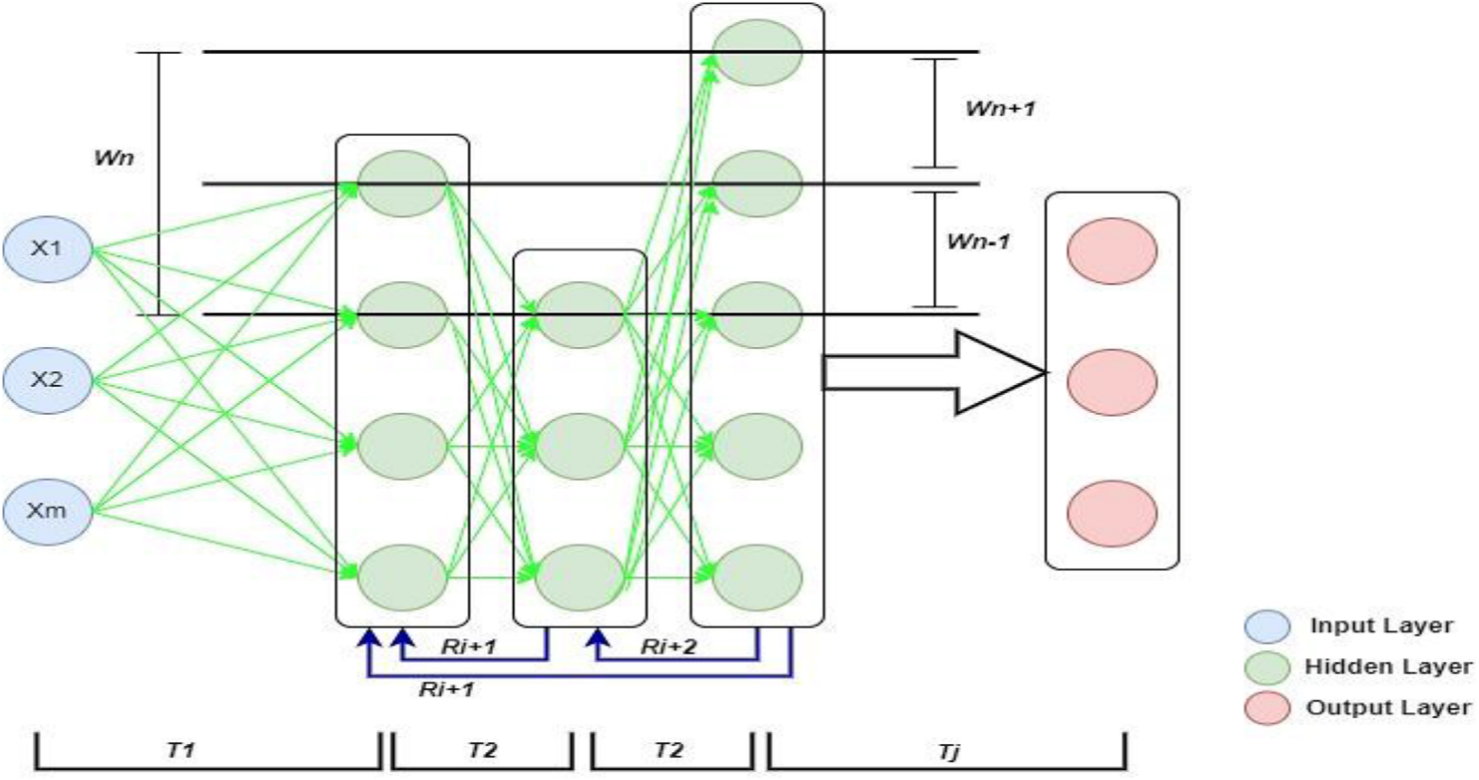
Proposed structure of neural network.

One of the most random operations that take place in the image encryption process is the process that accompanies the random division and segmentation of the image, in addition to the random distribution of pixels in the image through deep learning, consisting of several stages in the formation of the hidden layers of the neural network and the feedback process that allows reprogramming each of the hidden layers according to the infectiousness of each iteration. The well‐known standard neural network components are the main parts such as the input layer, the hidden layer, and the output layer. Deep learning contributes and interferes with the components of the hidden layer depending on the input layer, which is the main interest of the work. Several parameters control the deep neural network, and some of them are variables that can be changed according to the desired result, while some are fixed parameters that can only be changed by changing the structure of certain layer in the neural network. These parameters will be illustrated in details during the discussion of design neural network. Figure 5 shows the adaptive design of deep neural network.

where *w* considers the weight of neural network derived from each hidden layer such as hidden layer got variable length (number of nodes) from certain layer into next layer can get by:

(6) $$\;y_{m}=w_{nm}\;.x_{m}$$

This function is called transection function from layer to another layer (except for recursive flow), and source node is given by $x_{m}=x_{m-1}\;\mathrm{mod}x_{m}$ and destination node is given by $y_{m}=y_{m}\;+y_{m+1}$. The most important thing is how to control the recursive function that will be discussed in the next paragraph.

Output layer is considered the final result reflecting the complexity with desired results. Relevantly, hyperbolic equation reflects the result after many iterations in neural stage to achieve good prediction. Furthermore, neural machine can give many encryptions, and it is crucial to automatically to choose the best one to increase the efficiency of the proposed system. Hence, deep learning, namely machine learning was proposed. The next section will detail the contribution in this issue.

Many variables control the neural network and by training the network with these variables, the best possible prediction result can be controlled. Several features are extracted from the medical image in the algorithm. The feature that has the highest impact on the result is chosen, and the least effect is ignored, and then the work cycle is repeated. If the result continues to increase, the system automatically increases one hidden layer. If the result is stable, increment one node in the specified hidden layer, and so on. The main interests of parameters are weight, transection, recursive number and flow, number of iteration ratio with feedback, and acknowledgement from each neuron and layer. In order to find the high impact parameter, the structure of neural network must be integrated, and the network must be fully connected to achieve result of each stage with flow of information through these stages.

**FIGURE 6:**
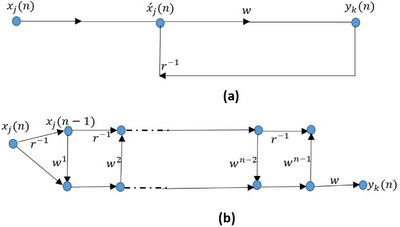
(a) signal flow graph of one stage in neural system; (b) flow graph with recurrent data flow of corresponding weight.

- The standard enclosure of neural system is represented by infinite weighted summation with two samples present and past for input signal (*x_j_* (*n*)) and delayed one is (*x_j_* (*n−*1)) represent delay by *1* time and the output signal *y_j_* (*n*) can be expressed with Equation (7):
(7) $$y_{k}\;\left(n\right)=\sum_{i=0}^{\infty }w^{i+1}x_{j}\left(n-1\right)$$

where *w* is the weight that controls the flow of data within the network, and *i* is the number of ` for training network. The structure is as illustrated in Figure 6.

In this regard, the weight derived from the structure achieves three cases distinguished as follows: (a) |w|<1 represents the output signal of $y_{k}\left(n\right)$ exponentially which means that the system is stable; (b) |w|=1 represents the linear behaviour; (c)|w|>1, represents negative exponential.

Now, for power *N*, the factor |w| will be small enough to achieve neglecting case as$w^{N}$, and for practical purpose, the situation produces the finite sum of $y_{k}$.

(8) $$y_{k}\left(n\right)\approx \sum_{l\;=\;0}^{N-1}w^{l+1}x_{j}\left(n-1\right)$$

This means that the suggested weight will be:

(9) $$\begin{array}{ccc} & & y_{k}\;\left(n\right)=wx_{j}\;\left(n\right)+w^{2}x_{j}\left(n-1\right)\\ & & +w^{3}x_{j}\left(n-2\right)+\cdots +w^{N}x_{j}\left(n-N+1\right) \end{array}$$

Then derived weight (*w*) will store in vector for next classification. Thus, in deep neural network, the weight will be changed according the behaviour of the system, whether it will increase or decrease for each iteration. In this study, weight parameter is classified in addition to other parameters to obtain the best prediction in the deep learning system.

- The flow of the system is the second parameter considered in the proposed system due to its direct effects on the result of next stage. There are many cases in the data flow such as when one input to the node produces one input, or two inputs from different nodes produce one output of the node, and many other cases for different iterations in the system. If input will classify into Boolean number such accepted or not in certain stage, and weight *w* consider the bias or control to the system in one stage and in one node.

This step will estimate the neurons in the next step based on the output function and bias that controls the weight at each stage.

**FIGURE 7:**
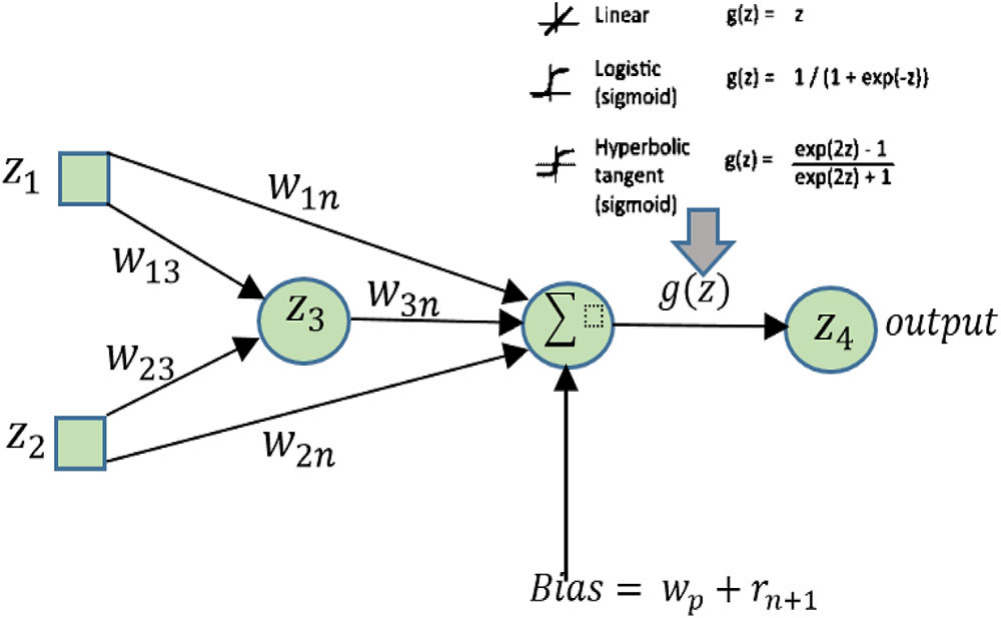
Strategy of node structure created and derivation.

Figure 7 illustrates the behaviour of neural when updating multiple layers in deep neural system whither forwarding or feedback data. XOR operation is implemented during iterations and in several ways. Direction of data flow comes from inherent node or not, and sometime from recurrent flow. All these give the factor for node to direct the new flow to the appropriate node.

The last pattern must give achieve $\mathrm{if}\;\left(w_{n}+w_{n+1}\right)>0\mathrm{then}\;x_{n+2}=\;1\;\mathrm{and}\;\mathrm{if}\;\left(x_{n}⊕x_{r(n+2)}\right)=\;1\;\mathrm{then}\;\mathrm{new}\;\mathrm{nod}\;\mathrm{created}$. Two patterns at the same layer cannot achieve the same result function because of the consistent update and control of data flow by predicted bias.

All transaction flow will store in certain vector to classify later as a deep learning system for better prediction.

- Recurrent network is the third parameter that can be used for deep neural network to increase accuracy. Recurrent network can be defined as the flow distinguishes from other feed‐forward neural layers and represented as at least one feedback loop.

## 4 RESULT AND DISCUSSION

In this section, the proposed algorithm for encryption and securing medical images was evaluated and tested. Two types of images namely colour and grey medical images were used in our study, and the test was also done on images from a standard dataset in order to benchmark and find out the strength of the proposed method. The image resolution was 512 × 512, and the result was simulated using MATLAB 2015a, a laptop computer equipped with CPU of Core i9, 3.9 speed, 32 GB memory, and Fedora 32 operating system. Among the information used in the algorithm is the size of the blocks in the partition which was 32 (and *n = *4) which was repeated 10,000 times. The proposed method is adapted to images with different dimensions, because it works on any distribution of pixels and any partition of the sub‐blocks. As for the segmentation process, it is not affected in any way, and the deep neural network works in the same way in different types of images.

### 4.1 Simulation results

Simulated results on image security refer to the use of computer simulations to evaluate the effectiveness of different techniques and algorithms for securing digital images. This process involves creating a digital image and then testing it against various attacks that can compromise its security, such as tampering, copying, or alteration. There are several techniques used in simulated image security testing, including watermarking, steganography, and encryption. In proposed method we consider encryption between two sides. Encryption is a technique that involves transforming the image data into a form that can only be decrypted by authorized parties. This can help to protect the image from being accessed or altered by unauthorized individuals.

**FIGURE 8:**
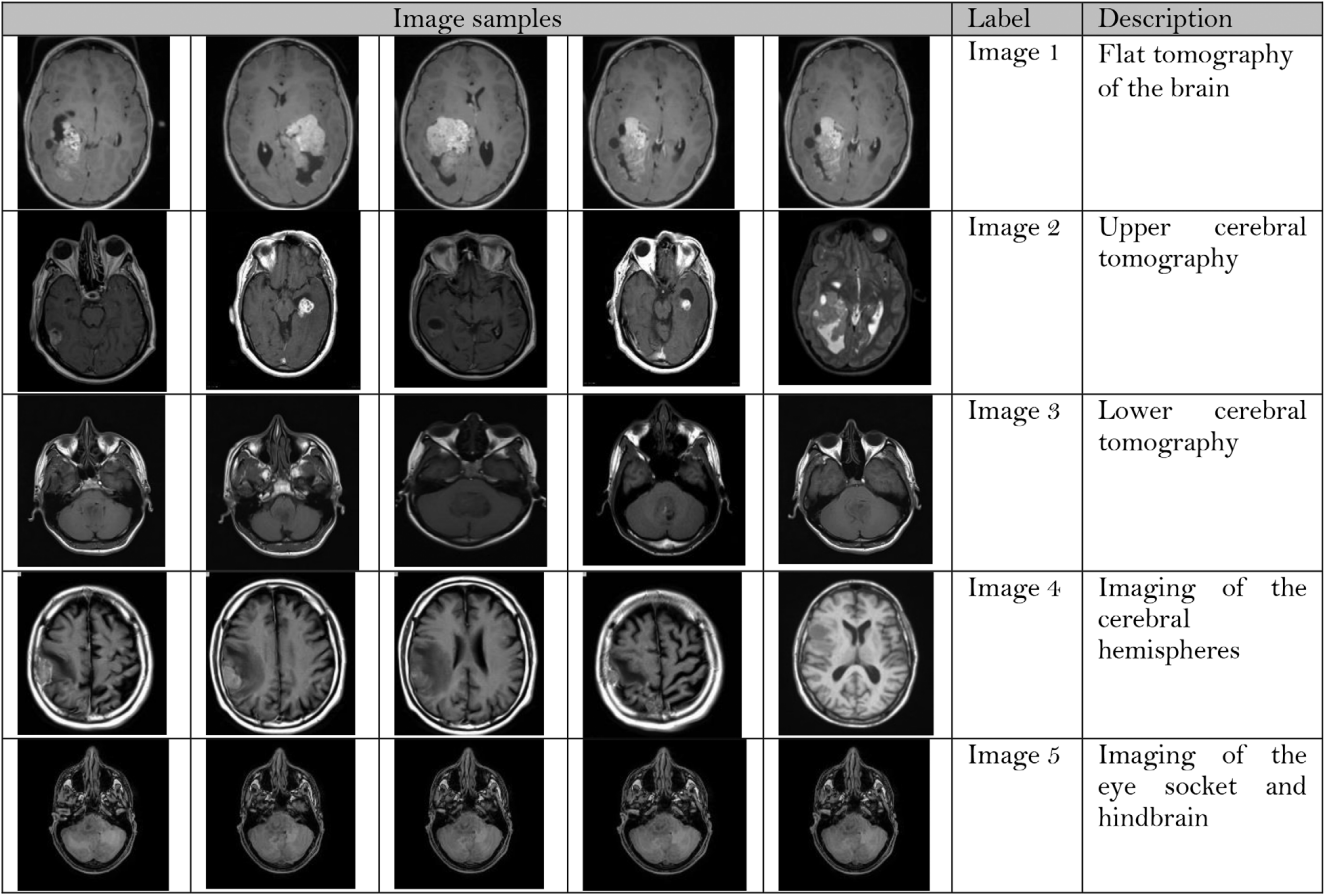
Labels of five class images used in evaluation the results.

Simulated results on image security are an important tool for evaluating the effectiveness of image security techniques and algorithms. They can help to improve the security of digital images and protect them from unauthorized access and tampering. Many images were considered in this study and most of these images are from a standard brain tomography dataset. Figure 8 depicts image labels that used in evaluation through proposed method within five classes.

In encryption, there are many evaluation criteria achieved such as:

i. information entropy

The randomness of the image can be measured in information entropy and is defined by the following equation:

(10) $$H\;\left(m\right)=\sum_{i=1}^{w}P\left(m_{i}\right)\mathrm{lo}g_{2}\frac{1}{P\left(m_{i}\right)}$$

For instance, *P*(*m*) considers the probability of the appearance *m*, to grey scale image where the max entropy will be 8. When the entropy reaches 8, it means that the randomness of the image is large and good, that is, the distribution of pixels on the image is more random. Table 1 shows the randomness of the encrypted medical image, where the entropy is close to 8.

**TABLE 1:** Entropy of proposed algorithm.

Tested images	Entropy
Image 1	7.9992
Image 2	7.9994
Image 3	7.9993
Image 4	7.9984
Image 5	7.9989

In this regard we can conclude that the proposed method is worthy in generated randomness of encrypted image.

ii. Image Histogram

**FIGURE 9:**
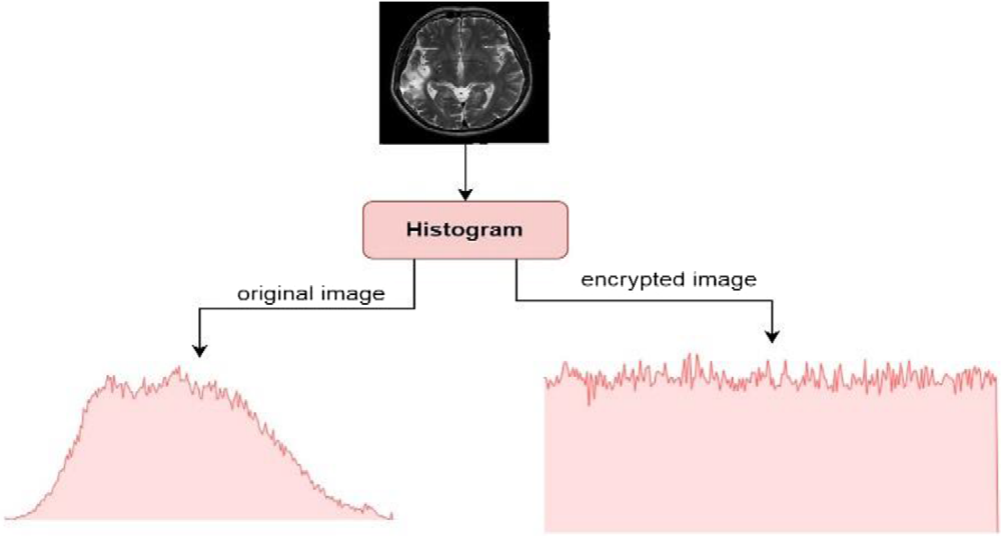
Histogram of pain image and encrypted image.

The histogram reflects the behaviour and distribution of the pixels in the image. In the encrypted image, the histogram should prevent attackers from guessing the image information. The histogram of the encrypted image should not be the same as the original image. In Figure 9, the graph shows a medical image before and after encryption.

The experiment further confirmed the histogram calculations of the encrypted image, which depend on the square test calculated as follows:

(11) $$\;X^{2}=\sum_{i=1}^{256}\frac{{\left(O_{i}-EV\right)}^{2}}{EV}$$

where *O_i_* is the recurrent rate for grey value *i*, and *EV is* O/256 is the expected frequency of the grey scale. Accordingly, the chi‐square of the encrypted image is illustrated in Table 2.

**TABLE 2:** Analysis of Chi‐Square of encrypted image.

Given image	Encrypted image
Image 1	260.9
Image 2	241.1
Image 3	231.5
Image 4	270.5
Image 5	212.8

Among the tests and evaluations of the encryption algorithm, Chi‐Square is used, which is considered one of the most important statistical tests to determine the similarity between the original and the encrypted image. This is because the expected image should be as close as possible to the original image, and the best measure for this prediction is the Chi‐Square algorithm. The application of this type of standard because it is the only one capable of distinguishing between noise and an encrypted image, because noise degrades the loss of image data, but an encrypted image cannot lose any type of data. It is considered one of the most important measures of randomness.

iii. Correlation coefficient

Neighbouring pixels in a normal image have a coherent correlation. On this basis, the low correlation between adjacent pixels can reflect the strength of encryption in the image. Mathematically, the correlation between two adjacent pixels is determined by the following equations:

(12) $$r\;\left(A,B\right)=\frac{E\left(\left(A-E\left(A\right)\right)\left(B-E\left(B\right)\right)\right)}{\sqrt{D\left(A\right)D\left(B\right)}}$$

(13) $$E\;\left(A\right)=\frac{1}{s}\;\sum_{i\;=\;1}^{s}A_{i}$$

(14) $$D\;\left(A\right)=\frac{1}{s}\;\sum_{i\;=\;1}^{s}{\left(A_{i}-E\left(A\right)\right)}^{2}$$

From the above: *A* and *B* are considered as the values of two adjacent pixels, where *s* is the number of selected pairs (*A* and *B*). The values of the correlation coefficient for the encrypted medical image are in the form of vertical *V*, horizontal *H* and diagonal *D* directions. The correlation coefficient in the images is close to zero and the correlation coefficient values are presented in Table 3. The evaluation also includes the benchmarking with existing method in literature as shown in Table 4.

iv. Differential attack

**TABLE 3:** Correlation coefficient of proposed method.

Image	Direction	Original image	Encrypted image
Image 1	*V*	0.987	−0.011
	*H*	0.976	0.019
	*D*	0.962	0.014
Image 2	*V*	0.991	0.005
	*H*	0.991	−0.007
	*D*	0.976	−0.041
Image 3	*V*	0.987	−0.022
	*H*	0.976	0.006
	*D*	0.962	0.008
Image 4	*V*	0.977	−0.041
	*H*	0.981	0.009
	*D*	0.976	0.004
Image 5	*V*	0.981	0.062
	*H*	0.973	0.081
	*D*	0.942	0.062

**TABLE 4:** Benchmarking of the correlation coefficient values with existing methods.

Method(s)	H	V	D
Proposed	−0.007	0.005	−0.041
[46]	−0.001	0.009	−0.003
[47]	0.002	0.001	0.001
[48]	0.002	−0.001	0.000
[49]	0.094	0.005	0.006
[17]	0.009	−0.007	0.018

Differential attack depends mainly on guessing information about the medical image by making a small change in the normal image and the encrypted image using the same method. The change should be so slight so that it does not arouse suspicion. To assess the performance, it is necessary to find the number of pixel change rate (NPCR) and the unified average changing intensity (UACI). Then, the Equation below is used:

(15) $$NPCR\;=\frac{1}{MN}\;\sum_{i\;=\;1}^{M}\sum_{j\;=\;1}^{N}D\left(i,j\right)\times 100\%$$

(16) $$D\;\left(i,j\right)=\left\{\begin{array}{c} 0\;\;\;if\;E_{1}\left(i,j\right)=E_{2}\left(i,j\right)\\ 1\;\;\;\;if\;E_{1}\left(i,j\right)\neq E_{2}\left(i,j\right) \end{array}\right.$$

(17) $$UACI\;=\frac{1}{MN}\;\sum_{i\;=\;1}^{M}\sum_{j\;=\;1}^{N}\frac{\left|E_{1}\left(i,j\right)-E_{2}\left(i,j\right)\right|}{255}\times 100\%$$

*E*_1_ and *E*_2_ are considered as two encrypted images of the plain and modified one (when changing one pixel in the plain image), while *M* is the width of image, and *N* is the height. Different attacks can be recognized by NPCR and UACI values as shown in Tables 5 and 6, respectively.

v. Key space

**TABLE 5:** Performances of NPCR and UACI.

Images	NPCR	UACI
Image 1	99.60	33.41
Image 2	99.61	33.40
Image 3	99.63	33.44
Image 4	99.61	33.45

**TABLE 6:** Performances of NPCR and UACI.

Methods	NPCR	UACI
Proposed	99.61	33.42
[46]	99.61	33.26
[47]	99.53	33.45
[48]	99.51	33.39
[49]	99.79	33.16
[17]	99.60	33.49

The key space is an important statistical standard and is used to measure the encrypted image, and it must be at least 2100, because key space lower than the aforementioned would mean that the algorithm is weak and can be broken using brute force attack. The initial condition *Y*_0_ can be a support for this method, while *a* is the control parameter, with iteration number *N*_0_ in chaotic map. Hence, the accuracy of *Y*_0_ will be 10^16^ while the *N*_0_ = 10^3^, and so, the total key space will be 10^35^. This will give the proposed algorithm the strength to stand against brute force attack.

## 5 LIMITATION

Each algorithm has limitations, including our proposed algorithm. These limitations can be summarized as follows: Computational load. Encryption algorithms, especially those that are concerned with images, are powerful, but require significant implementation time and effort, which constitutes a challenge in itself. Key management is considered important, especially after the development of information technology and the experiences gained by hackers and intruders. In the event that the encryption key is lost or cannot be accessed, it becomes impossible to decrypt the image, so in the future a recovery must be developed for such cases. Among the limitations is the incompatibility with compression or reduction and enlargement. In this case, the data that is inside the image may be lost and cannot be returned to. This creates a lot of complexity in such cases.

## 6 CONCLUSION

Medical images have recently become the subject of interest to many researchers, especially in keeping them secure. This has led to the need to use AI algorithms, in order to move away from the traditional methods to obtain better results. The deep neural network method was used to segment medical images, distribute the small parts randomly, and then distribute the pixels to certain areas randomly. Using the deep neural network technique, the randomness that was used in confusion and diffusion was increased. The deep neural network was developed by calculating the high weights that have an effect on each hidden layer of the system and returning the useful feedback to the nodes in those layers. One of the most important elements that have been taken care of in the proposed method is the process of distributing and dividing the image blocks according to the most impact variables in the result of the deep neural network. And in both stages, confusion, diffusion, and randomness of pixels in the image in additional to scrambling bits of pixels for changing pixel value. When evaluating the proposed algorithm by several criteria, as well as when benchmarking the results with previous research, the proposed method has been proven.

## AUTHOR CONTRIBUTIONS

All authors contributed equally to accomplish this research study.

## CONFLICT OF INTEREST STATEMENT

The authors declare no conflicts of interest.

## Data Availability

The data will be available upon request to the corresponding author.
